# Integrated Transcriptome and Metabolome Analysis of Color Change and Low-Temperature Response during Flowering of *Prunus mume*

**DOI:** 10.3390/ijms232112831

**Published:** 2022-10-24

**Authors:** Bin Dong, Zifei Zheng, Shiwei Zhong, Yong Ye, Yiguang Wang, Liyuan Yang, Zheng Xiao, Qiu Fang, Hongbo Zhao

**Affiliations:** 1 School of Landscape Architecture, Zhejiang Agriculture and Forestry University, Hangzhou 311300, China; 2 Zhejiang Provincial Key Laboratory of Germplasm Innovation and Utilization for Garden Plants, Hangzhou 311300, China; 3 Key Laboratory of National Forestry and Grassland Administration on Germplasm Innovation and Utilization for Southern Garden Plants, Hangzhou 311300, China

**Keywords:** *Prunus mume*, cold response, transcriptome, metabolome

## Abstract

In China, *Prunus mume* is a famous flowering tree that has been cultivated for 3000 years. *P. mume* grows in tropical and subtropical regions, and most varieties lack cold resistance; thus, it is necessary to study the low-temperature response mechanism of *P. mume* to expand the scope of its cultivation. We used the integrated transcriptomic and metabolomic analysis of a cold-resistant variety of *P. mume* ‘Meiren’, to identify key genes and metabolites associated with low temperatures during flowering. The ‘Meiren’ cultivar responded in a timely manner to temperature by way of a low-temperature signal transduction pathway. After experiencing low temperatures, the petals fade and wilt, resulting in low ornamental value. At the same time, in the cold response pathway, the activities of related transcription factors up- or downregulate genes and metabolites related to low temperature-induced proteins, osmotic regulators, protective enzyme systems, and biosynthesis and metabolism of sugars and acids. Our findings promote research on the adaptation of *P. mume* to low temperatures during wintering and early flowering for domestication and breeding.

## 1. Introduction

Low-temperature stress is the primary abiotic stress that plant species experience during their growth and development. Temperature is a major determinant of a plant’s geographical distribution, and temperature can induce significant changes in plant metabolism [1,2]. The response of plants to low temperatures can be complex, including temperatures above or below 0 °C, the exposure time, and the capability of cold acclimation [3]. Cold acclimation is the process induced in plants that enables them to increase their tolerance in response to low, non-freezing temperatures [4]. Cold exercise is a process in which plants are kept in a low-temperature environment for a period of time that does not cause serious injury but significantly improves resistance to low temperatures. During this process, many cold-related physiological indicators accumulate, and many protective proteins are induced [5,6]. When plants incur freezing stress, the interior of cells freezes, which destroys tissues, resulting in more serious damage than the cold exercise.

The response to cold stress can be divided into several steps: cold signal perception and reception, signal transduction, upstream and downstream gene expression response, and physiological and biochemical changes [7,8]. Response to low temperature is a multi-gene regulatory process, and the most significant breakthrough concerning this process is the discovery of the C-repeat (CRT) element in the promoter regions of cold-responsive genes and the corresponding C-repeat-binding factors (CBFs) [9,10]. The identification of CRT and CBFs has greatly facilitated the study of the gene expression regulatory networks in response to low temperatures [11,12].

In cold climates, plants have specialized mechanisms to survive. When plants sense low temperatures, transcription factors are activated to regulate the expression of a large number of cold-responsive genes (COR) [12,13]. The complex and intersecting metabolic pathways are activated to form a delicate set of resistance mechanisms to cold and freezing stresses, such as the ability of plants to cold exercise and recover from freezing [14,15]. The molecular mechanisms of plant response to low temperature have been well investigated for many plants [14,16]. Research on *Arabidopsis thaliana* provides considerable information for understanding the mechanism of low-temperature response, but the particular genetic backgrounds of different species and the interaction with the environment make understanding more complex [17,18]. For perennial herbs, temperature is the main trigger for cold exercise, and the rate of cold exercise is relatively fast, whereas the woody perennials are usually triggered by two incentives, low temperature and short photoperiod [19,20,21]. The response to low temperature involves distinct changes in gene expression, protein expression, and metabolites [22,23]. The trigger mode of low temperature and short photoperiod is also the main reason that woody plants have a significantly stronger exercise and tolerant ability toward low temperature than do perennial herbs. Although the phenotypes and physiology of responses of woody perennials to photoperiod and low temperature have been extensively reported, little is known at the molecular level about the response mechanisms.

In the prior two decades, advances in high-throughput sequencing and omics have facilitated the study of changes in gene expression, protein, and metabolites in response to various internal and external environments [24]. These technologies are effective means to comprehensively understand the response mechanism of cold stress in woody plants and increase the understanding of the signaling pathways and complex regulatory networks of the cold exercise process. On the basis of RNA-seq, investigators found that 10–15% of genes were differentially expressed in *Arabidopsis*, rice, and maize in response to low temperature [25,26,27]. In *Arabidopsis*, 306 COR genes were identified, 45 of which were regulated by CBF1 [28]. In woody plants, transcriptome profiling of grapefruit low-temperature tolerance revealed that transcripts related to photosynthesis, defense, cell wall, and secondary metabolism were downregulated, whereas transcripts related to membrane proteins, lipid metabolism, plant hormones, and cold-responsive transcription factors were upregulated [29]. Transcriptome sequencing of *Ammopiptanthus mongolicus* seedlings under cold acclimation detected many candidate genes and transcription factors differentially expressed in response to cold stress [30]. Transcriptome profiles of *Camellia sinensis* during cold acclimation identified differentially expressed genes in response to low and non-freezing temperatures, including a group of cold sensor or signal transduction genes, transcription factors, plasma membrane stabilization-related genes, and detoxification enzyme genes [31].

*Prunus mume* belongs to the Rosaceae family and is a famous traditional flowering tree in China. *P. mume* has a 3000-year history of cultivation [32]. Due to its high ornamental and economic value, *P. mume* has been widely cultivated in East Asia as well [33]. *P. mume* grows in tropical and subtropical regions. The plant has some degree of cold resistance, but most varieties essentially lack resistance, leading to the restriction of *P. mume* cultivation in Northern China where only a few varieties with strong cold resistance have been grown. Therefore, it is necessary to study the low-temperature response mechanism of *P. mume* to expand the scope of its cultivation.

In 2012, the entire genome of *P. mume* was sequenced [34], and 5.34 million SNP markers were mined by resequencing and genome-wide association studies of different *P. mume* varieties and related species. These analyses provide good genetic resources and a foundation for understanding flower color, flower fragrance, and stress resistance of *P. mume* [35,36]. Because of the rapid development of genomics and transcriptomic research on *P. mume*, investigators have comprehensively detailed the molecular mechanism of cold tolerance of *P. mume* and the related mechanisms of early flowering and dormancy in the ICE-CBF-COR pathway [34]. For example, the *PmCBFa*, *PmCBFb*, and *PmCBFc* genes were cloned from *P. mume*, and were found to be induced by low temperatures [37]. *P. mume* ‘Meiren’ has bright red leaves and purple branches, which are rare characteristics among other varieties that endow its high ornamental value [38]. *P. mume* ‘Meiren’ is a medium cold-resistant variety. It flowers from March to mid-April. Although it flowers later than other varieties, it is also vulnerable to cold waves during flowering. After a cold wave, *P. mume* ‘Meiren’ undergoes obvious changes in flower color and quality. However, there is a lack of research on the mechanism of its cold resistance and flower color changes. Thus, by integrating transcriptomic and metabolomic analysis, we identified key genes of *P. mume* ‘Meiren’ that respond to low temperatures. We propose a metabolic network for elucidating the response and adaptation of *P. mume* ‘Meiren’ to low-temperature stress and the mechanism of the low-temperature effect on flower color.

## 2. Results

### 2.1. Flower Phenotype under Low-Temperature Treatment

**Figure 1:**
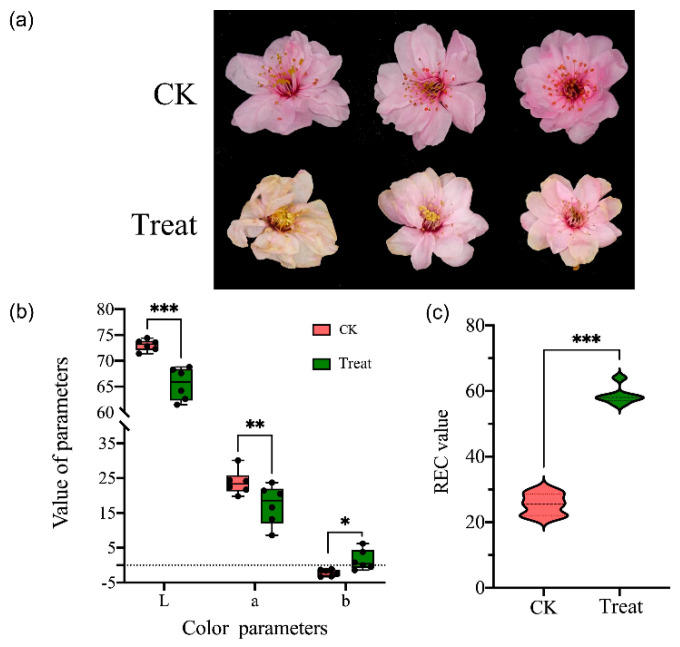
The morphology and color change of *Prunus mume* flowers in natural temperature (CK, 15 °C) and low-temperature treatment (−4 °C). (**a**) The morphology of *P. mume* flower of control and treatment groups. (**b**) The color parameters (CLE *Lab*) of petals of control and treatment groups, *L* is the lightness, *a* and *b* are two chromatic components measured. (**c**) The relative electrical conductivity (REC) of two groups. Significant differences were determined by Student’s *t* test, *p* < 0.05 (*), *p* < 0.01 (**), *p* < 0.001 (***).

The ornamental value of flowers can be negatively affected by low temperatures during flowering. Here, we measured the color parameters (L and C) and relative electrical conductivity (REC) to investigate the flower phenotype changes after low-temperature treatment. Compared with the control (normal temperature) group, the flower of *P. mume* ‘Meiren’ showed some wilting symptoms after low-temperature treatment (Figure 1a); the flower color became lighter, and the values of L and C were significantly decreased (Figure 1b). In addition, the value of REC rapidly increased immediately after low-temperature treatment (Figure 1c).

### 2.2. Overview of Transcriptome between Control and Low-Temperature Treatment Samples

**Figure 2:**
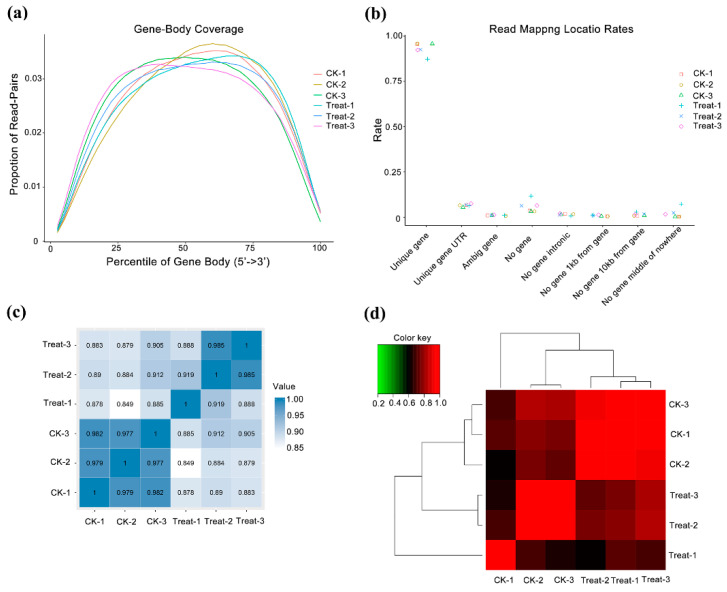
Quality control of transcriptome data and analysis of samples repeatability. CK means the control group (sampled at 15 °C), treat means the treatment group (sampled at −4 °C). (**a**) Randomness assessment of reads on reference genome. The abscissa is the relative position of the gene, the ordinate is the ratio of reads in the total aligned reads in the corresponding position interval, and different colors represent different samples. (**b**) Sample correlation analysis. This is a heatmap of the Pearson correlation coefficients between samples calculated based on all genes; the darker the color, the better the correlation between the two samples. (**c**) Cluster analysis between samples. (**d**) Sample correlation matrix analysis.

We constructed and sequenced six libraries and obtained 40 million paired-end reads in each library, with GC contents from 42.98–46.41%. The Q20 was 97.80% and Q30 exceeded 93.38% (Table 1). When single-ended clean reads were mapped to the reference genome of *P. mume*, the unique mapped reads occupied more than 84.03%, multiple mapped reads were between 3.48–3.50%, and unmapped reads were from 7.56–12.43% (Table 1). We identified 25,130 genes, qualified with FPKM, and annotated in databases of Uniprot, KEGG Pathway, and GO (Table S2). To evaluate the randomness of reads on reference genes, the positional distribution of reads on the gene reflects the coverage of the reads on the gene. Here, the reads evenly covered all positions of the full length of the gene (Figure 2a). On the basis of the annotation of gene structure, we mapped most sequences to the exon regions of unique genes, reflecting the high quality of transcriptome assembly (Figure 2b). For biological replication, we verified the reproducibility by sample correlation analysis and hierarchical clustering analysis. The sample correlation analysis showed that the three replicates of the two samples had a high degree of correlation, at more than 0.97 and 0.89 in control and low-temperature samples, respectively (Figure 2c). According to gene expression, we performed cluster analysis and found that the gene clusters with the same or similar expression patterns were included in different repeated samples, which also showed good repeatability in samples (Figure 2d).

### 2.3. Differentially Expressed Genes May Involve in Low-Temperature Response

**Figure 3:**
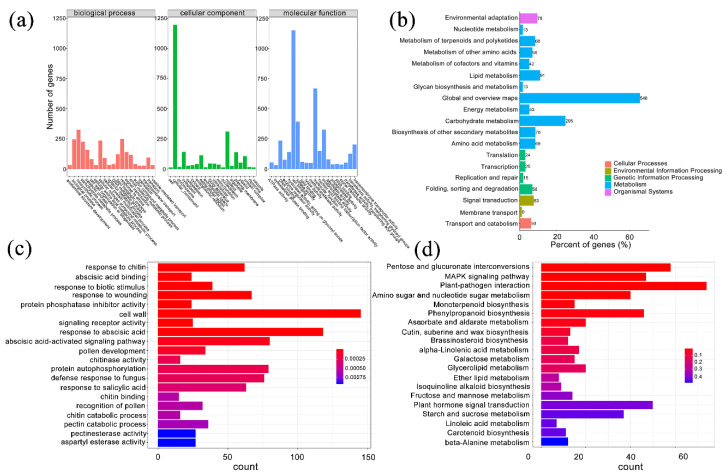
Functional classification and enrichment analysis of differentially expressed genes (DEGs) in *P. mume*. (**a**) Gene ontology (GO) classification of DEGs. The horizontal axis is the GO slim classification, and the vertical axis is the number of genes. (**b**) KEGG pathway classification of DEGs. The vertical axis is the name of the KEGG metabolic pathway, the horizontal axis is the ratio of the number of genes annotated to the pathway to the total number of annotated genes, and the value in the figure is the number of genes annotated to the pathway. (**c**) GO enrichment analysis of DEGs. The horizontal axis is the number of differentially expressed genes, and the vertical axis is the functional classification of GO. The color represents the *q*-value, and the smaller the *q*-value, the more significant it is. (**d**) KEGG enrichment analysis of DEGs. The horizontal axis is the number of differentially expressed genes, the vertical axis is KEGG Pathway, the color represents *q*-value, and the smaller the *q*-value, the more significant the enrichment result is.

By differentially expressed gene (DEG) analysis, we identified 4425 genes differentially expressed in two samples: 3247 of them were upregulated in the treatment group, and 1178 genes were upregulated in control group (Table S3). To characterize the function of these DEGs, we used the gene ontology (GO) annotation to classify them into three component categories. Each category contained 20 GO terms, and most terms contained fewer than 500 genes. Nonetheless, there were several terms that showed many genes. Biological process, cellular component, and ion binding had the most genes in categories of biological process, cellular component, and molecular function, respectively (Figure 3a). We found 1530 DEGs in 19 KEGG pathways that covered five classes, cellular processes, environmental information processing, genetic information processing, metabolism, and the organismal system. The five pathways with the greatest number of genes were global and overview maps, carbohydrate metabolism, lipid metabolism, biosynthesis of secondary metabolites, and signal transduction (Figure 3b). We performed GO and KEGG enrichment analysis to obtain genes with similar functions and identify DEGs associated with the most important biological processes, biochemical metabolic pathways, and signal transduction pathways. We found 1005 genes enriched in the top 20 GO terms; the term ‘cell wall’ was enriched with the most genes, followed by ‘response to abscisic acid.’ The top three terms with the most significant enrichment were ‘response to chitin’, ‘abscisic acid binding’, and ‘response to biotic stimulus’ (Figure 3c). In KEGG enrichment, the top 20 pathways contained 418 DEGs, and the most significant pathways were ‘plant-pathogen interaction’, ‘pentose and glucuronate interconversions’, ‘MAPK signaling pathway’, ‘phenylpropanoid biosynthesis’, and ‘amino sugar and nucleotide sugar metabolism’ (Figure 3d).

### 2.4. The Major Regulator Genes Response to Low Temperature in P. mume ‘Meiren’

The ICE-CBF-COR is the most important low-temperature response pathway in plants. We classified the core genes that direct the response to low temperatures. After the flower buds experience low temperatures, the components that showed the highest expression level were the DREB/CBF transcription factor, ICE1 transcription factor, cold-responsive protein kinase, low-temperature-induced protein, and late embryogenesis (Table 2). In addition, we identified major transcription factors (TFs) and genes differentially expressed following cold treatment.

**Figure 4:**
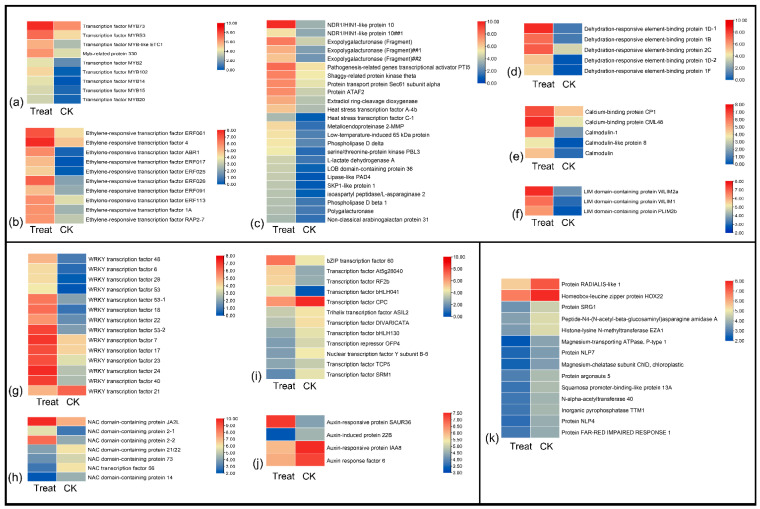
The heatmap of major regulator genes response to low temperatures in *P. mume.* (**a**–**f**) Upregulated genes after cold stress, including MYB family, ethylene-responsive transcription factors, dehydration-responsive element-binding proteins, calmodulin-related proteins, LIM domain-containing proteins, and others may involve in cell processes and biosynthesis. (**g**–**j**) Up- and downregulated genes after cold stress. (**k**) Downregulated genes response to low temperatures.

There were several types of TFs and genes that upregulated after low-temperature exposure, including MYB (Figure 4a) and ethylene-responsive transcription factors (Figure 4b), dehydration-responsive element-binding proteins, calmodulin-related proteins, LIM domain-containing proteins, and others involved in cell processes and biosynthesis (Figure 4c–f). The major members in the WRKY family were upregulated after low-temperature treatment, but WRKY21 was downregulated (Figure 4g). In the NAC family, the NAC domain-containing proteins JA2L and 2 had the highest expression level in the treatment sample, but NAC56 and other NAC domain-containing proteins were downregulated after low-temperature treatment (Figure 4h). In addition, the bZIP60, RF2b, and bHLH041 TFs showed high expression in the treatment group; conversely, CPC, bHLH130, NF-YB5, and SRM1 were upregulated in the natural temperature control group (Figure 4i). In addition, the auxin-related factors also showed different expression levels; only the auxin-responsive protein SAUR36 was upregulated after low-temperature treatment, others were downregulated, including the auxin-induced protein 22B, auxin-response protein IAA8, and auxin-response factor 6 (Figure 4j). Figure 4k present mainly low-temperature downregulated genes, such as SRG1, NLP7, and NLP4.

### 2.5. Metabolite Profiles of Prunus mume in Response to Low Temperature

**Figure 5:**
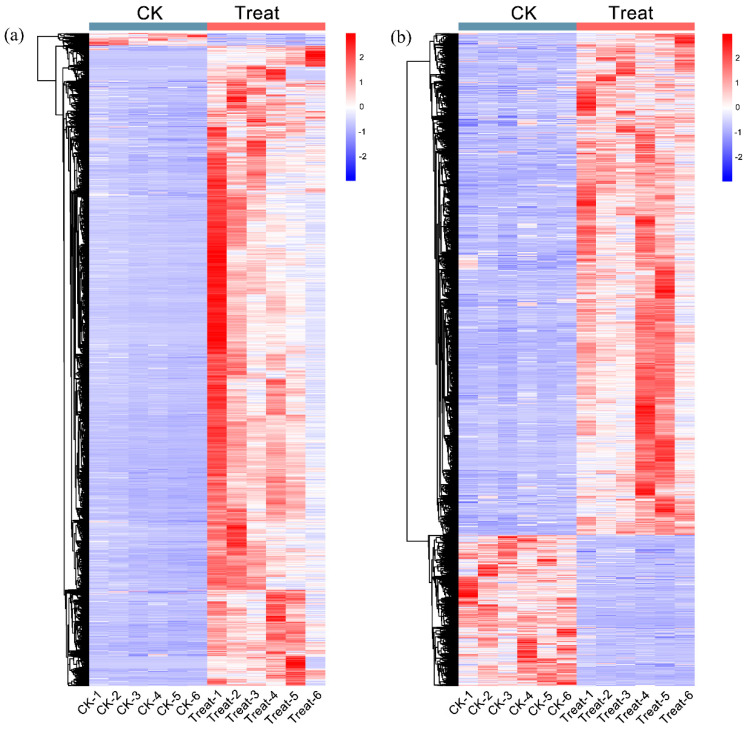
The differential metabolite hierarchical clustering analysis. (**a**) The differential metabolite heatmap of a positive ion. (**b**) The differential metabolite heatmap of negative ion. The magnitude of the relative amounts is shown by the difference in color, where the columns represent the samples, and the rows represent the metabolites. CK means the control group (sampled at 15 °C), treat means the treatment group (sampled at −4 °C).

Mass spectrometry scans of the components separated by chromatography revealed two base peak chromatograms, the positive and negative ion modes (Figure S1a,b). To detect the different metabolites, all metabolite profiles were performed by quality control (QC), quality assurance (QA), and hierarchical cluster analysis. Principal component analysis showed that the QC samples were all densely distributed in both positive and negative ion modes (Figure S2a,c). In the QC samples, the proportions of characteristic peaks with RSD < 30% were 92.3% and 81.7% (Figure S2b,d) in two models, indicating the high quality of the data. The differential metabolite hierarchical clustering analysis showed that the metabolic levels of positive and negative ion models were significantly different between control and low-temperature samples (Figure 5). The primary filter of all metabolites showed 2684 upregulated metabolites, 287 positive and 2397 negative ions, in the control sample. In the low-temperature treatment sample, the upregulated number of metabolites was 22,852, with 14,833 positive and 8019 negative ions (Table S4). Most metabolites showed high metabolic levels in plants subjected to low temperatures, which indicated that low temperature greatly affected the metabolite content of *P. mume*. In all detected ions, 3452 metabolites were annotated (Table S5), including 848 secondary metabolites (Table S6).

### 2.6. Differentially Accumulated Metabolites (Dams) in Response to Low Temperature

Among the annotated metabolites, there were 451 that showed significant differences in fold change after low-temperature treatment (Table S7). The top five significantly increased metabolites were acetoacetic acid, succinic acid semialdehyde, 2-furancarboxaldehyde, isochavicol, and phosphorylcholine. Conversely, the metabolites with significant reductions were phenylethylamine, sorbitol, ethyl methyl acetic acid, L-beta-phenylalanine, and L-leucine (Table S7). These differential metabolites are involved in 86 KEGG pathways (Table S8). In each pathway, we counted the total number of metabolites and the number of differential metabolites. There were 12 pathways enriched in more than 10 differential metabolites. In 4 of these 12 pathways, the number of differential metabolites exceeded 25% of the total. There were metabolites in flavonoid biosynthesis, flavone and flavonol biosynthesis, lysine biosynthesis, and linoleic acid metabolism (Table S8).

### 2.7. Correlation Analysis between DEGs and DAMs

**Figure 6:**
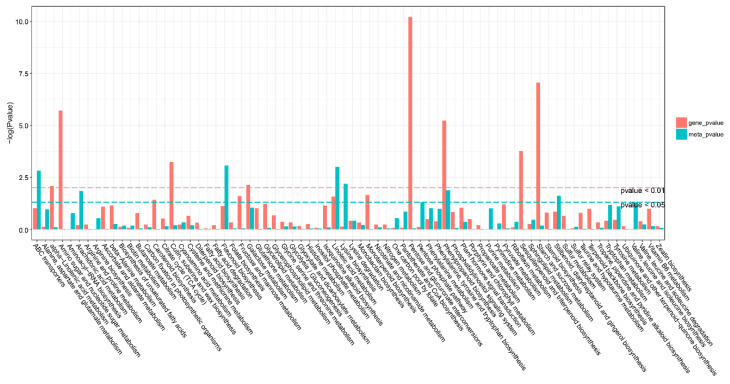
KEGG pathway analysis of differentially expressed genes (DEGs) and differentially accumulated metabolites (DAMs). The horizontal bar is the KEGG pathway, the vertical bar is the enrichment −log10 (*p*-value) value, the red in the ordinate represents the enrichment −log10 (*p*-value) value of differential genes, the green represents the enrichment −log10 (*p*-value) value of differential metabolites, the vertical axis. The higher the coordinate, the smaller the *p*-value value, and the stronger the enrichment.

**Figure 7:**
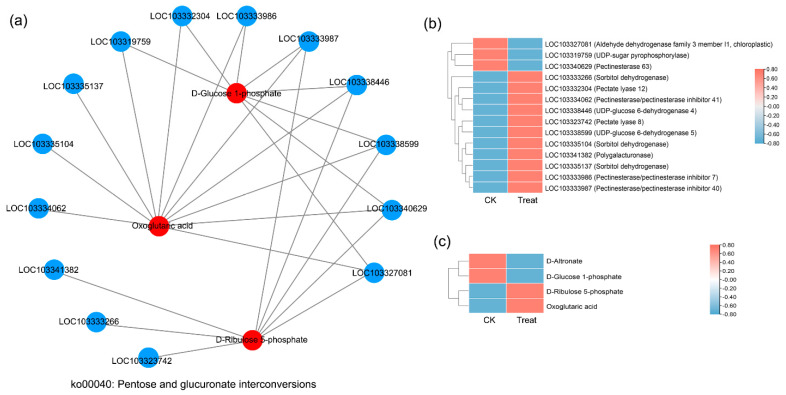
Correlation network diagram of the ko00041 pathway (pentose and glucuronate interconversions). (**a**) The correlation network diagram. Red color represents the enriched differential metabolites in the pathway, blue represents the differential genes enriched in the pathway, and the straight line represents the correlation between them. The higher the degree of the node, the more related straight lines, the node is considered more important. (**b**) The heatmap shows the expression level of correlated genes. (**c**) The heatmap shows the accumulation level of correlated metabolites.

**Figure 8:**
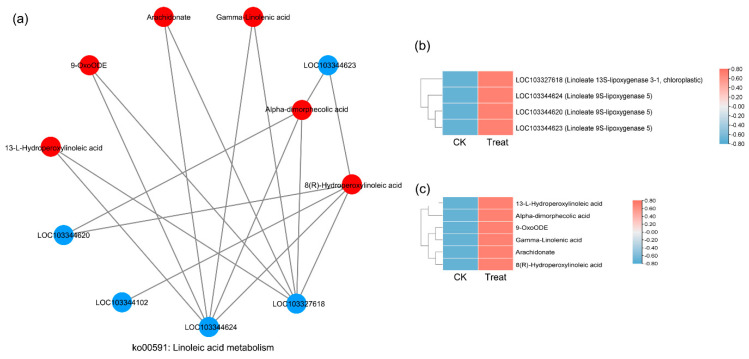
Correlation network diagram of the ko00591 pathway (linoleic acid metabolism). (**a**) The correlation network diagram. (**b**) The heatmap shows the expression level of correlated genes. (**c**) The heatmap shows the accumulation level of correlated metabolites.

There were 74 KEGG pathways with enrichment for both DEGs and DAMs (Figure 6). The DEGs in eight pathways reached extreme enrichment, including ‘pentose and glucuronate interconversions’, ‘starch and sucrose metabolism’, ‘amino sugar and nucleotide sugar metabolism’, ‘phenylpropanoid biosynthesis’, ‘sesquiterpenoid and triterpenoid biosynthesis’, ‘cutin, suberin, and wax biosynthesis’, ‘galactose metabolism’, and ‘alpha-linolenic acid metabolism’. However, the pathways enriched in DAMs were totally different from DEG enrichment. The DAM-enriched pathways were ‘flavonoid biosynthesis’, ‘linoleic acid metabolism’, ‘ABC transporters’, and ‘lysine biosynthesis’ (Figure 6). On the basis of the correlation analysis and KEGG enrichment pathways of genes and metabolites, we constructed the correlation networks for the enriched DEGs and DAMs that may be involved in cold stress. In the ko00040 network, pentose, and glucuronate interconversions (Figure 7), 14 DEGs and 4 DAMs were connected and showed different expression patterns. After cold stress, three genes related to aldehyde dehydrogenase, UDP-sugar pyrophosphorylase, and pectinesterase were downregulated, but others related to pectate lyase, pectinesterase/pectinesterase inhibitor, sorbitol dehydrogenase, and UDP-glucose 6-dehydrogenase were upregulated (Figure 7b). Correspondingly, D-altronate and D-glucose 1-phosphate were decreased after cold stress, but D-ribulose 5-phosphate and oxoglutaric acid accumulated (Figure 7c). In the network of ko00591, linoleic acid metabolism, (Figure 8), both metabolites (*p*-value < 0.01) and genes (*p*-value < 0.05) were enriched. There were four key genes and six metabolites upregulated in response to low temperature, and they together contributed to the high level of linoleic acid metabolism. The genes were linoleate 13S-lipoxygenase and linoleate 9S-lipoxygenase (Figure 8b), and the metabolites were 13-L-Hydroperoxylinoleic acid, gamma-linolenic acid, 8©-hydroperoxy linoleic acid, arachidonate, alpha-dimorphecolic acid, and 9-OxoODE (Figure 8c).

### 2.8. qRT-PCR Validation

**Figure 9:**
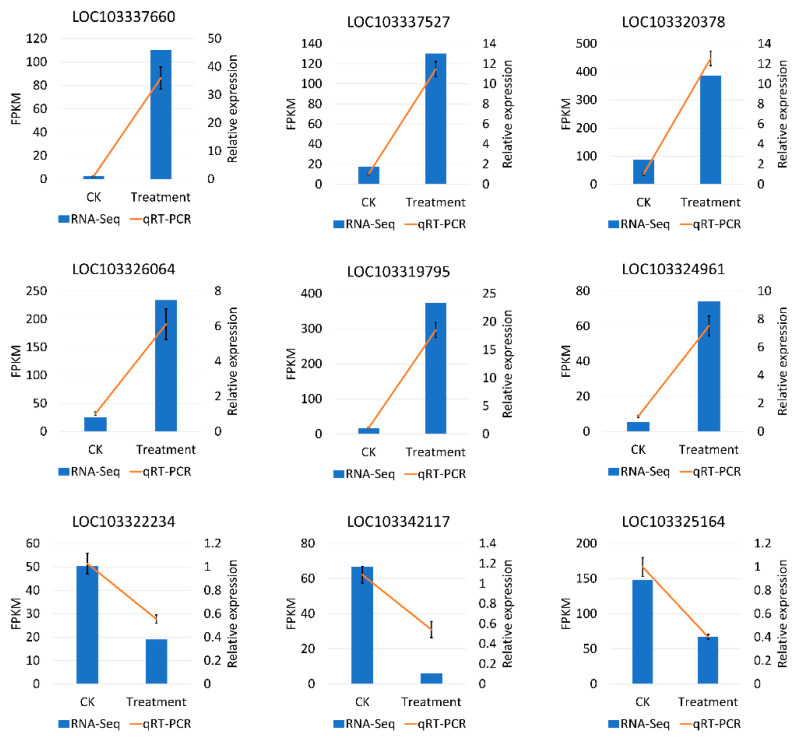
qRT-PCR validation of the expression level of nine DEGs may respond to the low temperature during flowering in *P. mume*. The *x*-axis means the two groups in this study, and the *y*-axis means the FPKM by RNA-Seq and the relative quantitative expression level by qRT-PCR.

To assess the reliability of RNA-Seq, we randomly selected nine DEGs that responded to low temperatures for qRT-PCR validation. The genes were WRKY53, WRKY7, MYB73, ethylene-responsive transcription factor 4, NAC2, NAC56, CBL-interacting protein kinase 5, zinc finger protein CONSTANS-LIKE 15, and auxin response factor 6. The expression patterns of these genes indicated a high degree of consistency between RNA-Seq and qRT-PCR, which indicated the reliability of the transcriptome (Figure 9).

## 3. Discussion

Under low temperatures, plants can improve cold tolerance by inducing or repressing gene expression [39]. With the release of the *P. mume* reference genome [34], the integrated analysis of transcriptomics and metabolomics reveals more about the cold response. In this study, the key genes and metabolites that responded to low temperatures during the flowering stage of *P. mume* ‘Meiren’ were screened by transcriptomics and metabolomics after low-temperature treatment of floral buds. There were 4425 differentially expressed genes, of which 3247 were upregulated after cold stress. Some of these DEGs were further classified as the core regulatory component that responds to low temperatures during the flowering period of *P. mume* ‘Meiren’, which provided a basis for subsequent gene function identification. When plants experience low temperatures, many inactive proteins and metabolites accumulate, and by a series of cellular responses and molecular strategies they stimulate powerful defense and immunity to abiotic stress [40]. When the flower bud of *P. mume* ‘Meiren’ experienced low temperatures, the metabolites accumulated differently compared with the control group. The identified metabolites and correlation analysis with DEGs help to understand the mechanism of low-temperature response, breeding, and use of cold-resistant varieties.

When plants experience low temperatures, Ca^2+^ acts as an important secondary signal molecule for sensing the changes in the environment. Calcium enters the cell through calcium ion channels and regulates gene expression, thereby regulating the response to various abiotic stress signals [41]. In this study, the expression of genes encoding calcium-binding proteins and calmodulin (CaM) were upregulated after low-temperature stress (Figure 4e). This finding suggested that extracellular calcium ions may rapidly influx under the stimulation of a cold environment, and the free calcium ions may activate calcium-binding proteins and calmodulin activity to signal low-temperature stress. This pattern has been demonstrated in other species [42,43]. After the cold stimulation signals are received, genes encoding stress-related proteins are expressed; these genes are mainly involved in pathways such as the dehydration process and sugar transport or decomposition. In *P. mume*, the genes related to the dehydration process (*PmLEAs*; Table 2) and glucose metabolism (pentose and glucuronate interconversions pathways; Figure 7) were highly expressed after low-temperature stress. Metabolome enrichment confirmed that low temperatures promoted the accumulation of linoleic acid, flavonoid, and lysine in *P. mume* (Figure 8). They may regulate the accumulation of downstream metabolites by the high expression of functional genes to improve cold resistance, which is important for plants to adapt to low temperatures.

Transcription factors have important functions in plant growth, development, and stress tolerance. Transcription factors regulate the expression of genes by interacting with cis-acting elements in the promoter regions of genes [44]. The TF families associated with plant stress tolerance mainly include AP2/EREBP, MYB, WRKY, and bHLH [45]. Many TFs are highly expressed when plants are subjected to low temperatures. For example, the banana fruit NAC transcription factor is involved in cold stress by interacting with MaCBF1. Transcription factor ZmMYB31 positively regulates the CBF genes to enhance the resistance to low temperatures [46]. Some WRKY TFs were induced during the cold acclimation stage in *Coffea canephora* [47] and *P. mume* [48]. In this study, the TF families of MYB (2, 14, 15, 20, 73, 102), ERF, WRKY (6, 7 17, 18, 22, 23, 24, 28, 40, 28, 53), NAC2, bZIP60, and bHLH041 were highly expressed after low-temperature stress (Figure 4). This high expression indicated that these TFs may be involved in the sensing and transcriptional response of low-temperature signals, and the corresponding genes may participate in the molecular regulation of cell reconstruction, tissue growth, and development in *P. mume*. There are 58 WRKY transcription factors in *P. mume*, and 12 of them showed significantly high expression after low-temperature treatment [48].

In many plants, the ICE-CBF-COR signaling pathway is the most important pathway that responds to cold stress. The pathway is regulated by CBF/DREB transcription factors, and it induces low-temperature tolerance [48,49]. Many genetic and molecular analyses have identified the C-repeat/DREB binding factors (CBFs) as key transcription factors that function in cold acclimation and are critical for cold acclimation in higher plants [50]. CBF has been isolated from many species, such as maize [51], rice [52], wheat [53], and tomato [54], which suggests that CBF is conserved. Overexpression of CBF leads to the induction of COR expression and increased frost resistance in many plant species [55,56]. In this study, the DREB/CBF transcription factors, ICE1 transcription factor, cold-responsive protein kinase, and temperature-induced protein were highly expressed after cold stress (Table 2), which not only confirmed the universality of the ICE-CBF-COR signaling pathway in response to low temperatures, but also contributed to the in-depth study of the molecular mechanism of this pathway to mediate cold tolerance of *P. mume*.

## 4. Materials and Methods

### 4.1. Plant Materials and Treatments

*P. mume* ‘Meiren’ was planted at Zhejiang Agriculture and Forestry University (30.26° N, 119.73° E). In March, three triennial potted plants with flowers were used for treatment at −4 °C in incubators with a 12 h light/12 h dark cycle and 50–60% relative humidity. Meanwhile, three triennial potted plants were set for the control check (CK) group under the natural temperature of 15 °C (same humidity as the treatment group). After 48 h, about 0.5 g flowers were collected for transcriptome sequencing and metabolomics analysis; three and six biological replicates were performed, respectively. At the same time, the flower color and relative electrical conductivity (REC) were determined under −4 and 15 °C conditions. The color parameters (CLE Lab) of petals including lightness (*L*), and two chromatic components *a*^∗^ and *b*^∗^ were measured by a Minolta CR-10 portable colorimeter (Konica Minolta, Japan) according to Wang et al. [57]. The flower chroma (*C*) was calculated by the formula *C* = (*a*^2^ + *b*^2^)^1/2^. Six measurements of each sample were performed. Then, 0.2 g petals were cut into pieces for determining the initial conductivity using Thunder Magnetic Conductivity Instrument (DDS-11A, Shanghai, China) after 4 h in ddH2O. The treated samples were boiled for 30 min and stood for 12–15 h to determine the final conductivity. The conductivity value of the same amount of deionized water was taken as the control. The electrolyte extravasation rate = [(initial conductivity − CK)/(final conductivity − CK)] × 100%. The biological repeats were performed three times.

### 4.2. RNA Extraction, Sequencing, and Analysis

The total RNA of flowers was isolated using an RNA extraction kit according to the protocol (Huayueyang, Beijing, China). The RNA quantity was measured by a Qubit RNA Assay Kit and Qubit 2.0 Fluorometer, the integrity was measured with Agilent Bioanalyzer 2100 system, and purity was determined with a NanoPhotometer spectrophotometer (IMPLEN, Westlake Village, CA, USA). Six cDNA libraries were generated using quality RNA with a NEBNext UltraTM RNA Library Prep Kit for Illumina (NEB, Ipswich, MA, USA). After fragments were purified and enriched, cDNA with the size 200–250  bp was used to construct the final cDNA library and sequenced at the Illumina HiSEquation 2000 platform in Benagen company (Wuhan, China). The raw sequence reads were processed into clean reads by filtering low-quality reads. The clean reads were mapped to the *P. mume* genome using the alignment software STAR (version: 2.7.0.d) [58]. The reference genome was downloaded from https://www.ncbi.nlm.nih.gov/genome/?term=Prunus+mume, accessed on 20 August 2021. According to the alignment files, the Qsort2 was used to perform statistics and graphs to evaluate the quality of the sequencing data, including the alignment rate, alignment region, and gene region coverage. The alignment results were counted using HTSeq (version: 0.11.2) [59] to obtain the number of reads aligned to each gene in each sample. The gene expression level was analyzed by FPKM (expected number of fragments per kilobase of transcript sequence per millions of base pairs sequenced) that was corrected by units of TMM [60]. On the basis of the expression levels of all genes (reads count) in each sample, we performed differential expression analysis using the software DESeq2 [61], with the screening threshold *q*-value < 0.05 and |log2FoldChange| > 1. The differentially expressed genes were subjected to enrichment analysis, namely, gene ontology (GO) with the GOseq R package [62] and KEGG (Kyoto Encyclopedia of Gene and Genomes) pathways with the KOBAS program [63].

### 4.3. Metabolite Extraction and Detection

Metabolite extraction was performed as follows: Each sample of 100–200 mg was put in a 2 mL EP tube, and then 0.6 mL 2-chlorophenylalanine (4 ppm) methanol (−20 °C) was added in it and the tube was vortexed for 30 s. Then, the glass beads of 100 mg were added for grinding for 90 s at 55 Hz into the tissue grinder. Then the mixture was examined via ultrasound for 15 min at room temperature, and centrifugated at 12,000 rpm at 4 °C for 10 min. The 200 μL supernatant was taken and filtered through a 0.22 μm membrane. The filtered sample was collected into the detection bottle for further detection. In each sample, 20 μL was taken for the quality control (QC) samples. Finally, the prepared samples were used for LC-MS detection [64,65].

Chromatographic separation was accomplished in a Thermo Vanquish system equipped with an ACQUITY UPLC^®^ HSS T3 (150 × 2.1 mm, 1.8 μm, Waters, Indianapolis, IN, USA) column maintained at 40 °C. The temperature of the autosampler was 8 °C. The gradient elution of analytes was conducted with 0.1% formic acid in water (A2) and 0.1% formic acid in acetonitrile (B2) or 5 mM ammonium format in water (A3) and acetonitrile (B3) at a flow rate of 0.25 mL/min. Samples of 2 μL of each sample were injected after equilibration. An increasing linear gradient of solvent B2/B3 (*v*/*v*) was used as follows: 0~1 min, 2% B2/B3; 1~9 min, 2%~50% B2/B3; 9~12 min, 50%~98% B2/B3; 12~13.5 min, 98% B2/B3; 13.5~14 min, 98%~2% B2/B3; 14~20 min, 2% B2-positive model (14~17 min, 2% B3-negative model).

The ESI-MSn experiments were executed on the Thermo Q Exactive HF-X mass spectrometer with the spray voltage of 3.5 and −2.5 kV in positive and negative modes, respectively. Sheath gas and auxiliary gas were set at 30 and 10 arbitrary units, respectively. The capillary temperature was 325 °C. The analyzer scanned a mass range of *m*/*z* 81–1000 for a full scan at a mass resolution of 60,000. Data-dependent acquisition (DDA) MS/MS experiments were performed with an HCD scan. The normalized collision energy was 30 eV. Dynamic exclusion was implemented to remove some unnecessary information in MS/MS spectra.

### 4.4. Metabolomics Data Analysis

The raw data were converted by ProteoWizard software (v3.0.8789), and then the peak identification, filtration, and alignment were performed using the XCMS package of R (v3.3.2). The main parameters were bw = 2, ppm = 15, peakwidth = c (5, 30), mzwid = 0.015, mzdiff = 0.01, method = centWave. The data matrix was obtained including the mass-to-charge ratio (*m*/*z*), retention time (rt), and peak area (intensity), and then the precursor molecules in positive and negative ion modes were generated. On the basis of the precursor molecules, we performed agglomerate hierarchical clustering analysis with the pheatmap package in R (v3.3.2) to determine the metabolic patterns of metabolites under different experimental conditions. To further mine the information, the ropls package of R was used for multivariate statistical analysis, including principal component analysis (PCA), partial least squares–discriminant analysis (PLS-DA), and orthogonal partial least squares discriminant analysis (OPLS-DA).

### 4.5. Differential Metabolite Identification and Correlation Analysis

Differential metabolites (biomarkers) were identified with the following criteria: *p*-value ≤ 0.05 and VIP ≥ 1. The exact molecular weight of the metabolites (molecular weight error < 30 ppm) was first confirmed, and, based on the fragmentation information obtained in the MS/MS mode, the accurate information of the metabolites was obtained by matching annotations in multiple databases, including Metlin (http://metlin.scripps.edu, accessed on 12 March 2022), massbank (http://www.massbank.jp/, accessed on 12 March 2022), LipidMaps (http://www.lipidmaps.org, accessed on 12 March 2022), and mzclound (https://www.mzcloud.org, accessed on 12 March 2022). Then we performed statistical analysis of metabolites, hierarchical clustering, correlation analysis, and pathway analysis of metabolites.

In the correlation analysis, we compared the KEGG pathways for enrichment of differentially expressed genes (DEGs) and differentially accumulated metabolites (DAMs) (*p*-value < 0.01 and 0.05), and then the enrichment was plotted using the R package. Pearson correlation coefficients of genes and metabolites were calculated using the scipy module (version: 1.3.1) in the software python. It is generally considered that the genes and metabolites were highly correlated when the Pearson correlation coefficient is greater than 0.8 and the *p*-value is less than 0.05. Finally, the DEGs and DAMs with high correlation in the KEGG pathway (*p*-value < 0.05) were selected to show the correlation by network diagrams created with Cytoscape [66].

### 4.6. Quantitative Real-Time Polymerase Chain Reaction Validation

To verify the reliability of RNA-seq, 16 DEGs involved in cold response were selected for qRT-PCR. The primer pairs (Table S1) were designed with the Primer 5 program (version 5.0, Premier Biosoft International, Palo Alto, CA, USA), and PP2A-1 was selected as the internal standard control [67]. Total RNA was used for first cDNA strand synthesis with the PrimeScriptTM reagent kit (Takara, Dalian, China). The mRNA expression was quantified using the SYBR Premix Ex Taq II kit (TaKaRa, Dalian, China). A quantitative real-time polymerase chain reaction (qRT-PCR) was conducted on a StepOnePlus™ real-time PCR system (Applied Biosystems, Foster City, CA, USA). Three biological and technical replicates for each gene were employed. Relative gene expression levels were calculated using the 2^−ΔΔCT^ method [68].

## 5. Conclusions

We used an integrated transcriptome and metabolome approach to identify the mechanism of low-temperature response in early spring flowering by *P. mume* cultivar “Meiren”. The cultivar “Meiren” can respond in a timely manner to temperature change by a low-temperature signal transduction pathway, and then induce related transcription factors that up- and downregulate specific genes that lead to the accumulation of related metabolites. These genes and metabolites include low-temperature-induced proteins, osmotic regulators, protective enzyme systems, biosynthesis, and metabolism of sugars and acids. Our findings promote research on the adaptation of *P. mume* to low temperatures during wintering and early flowering for domestication from south to north.

## Figures and Tables

**Table 1:** The statistics of transcriptome profile and the mapping result.

Sample	Total Reads	Total Bases	GC Content	Q20	Q30	Unique Mapped	Multiple Mapped	Unmapped
CK-1	41,384,788	6,207,718,200	43.91%	98.02%	93.81%	85.81%	3.5%	10.69%
CK-2	41,912,424	6,286,863,600	42.98%	97.91%	93.46%	84.43%	3.48%	12.09%
CK-3	42,921,588	6,438,238,200	44.79%	97.82%	93.38%	84.08%	3.49%	12.43%
Treat-1	43,484,436	6,522,665,400	46.41%	97.80%	93.42%	87.45%	4.99%	7.56%
Treat-2	40,726,270	6,108,940,500	45.46%	97.99%	93.90%	86.89%	4.91%	8.2%
Treat-3	42,130,680	6,319,602,000	45.04%	98.00%	93.85%	86.55%	4.82%	8.63%

CK: the control group (sampled at 15 °C). Treat: the treatment group (sampled at −4 °C).

**Table 2:** The core genes related to cold stress in *P. mume*.

Classification	Locus	Protein Name	Up/Down
DREB/CBF transcription factor	CBF	dehydration-responsive element-binding protein 1D	up
	CBF_1	dehydration-responsive element-binding protein 1D	up
	LOC103344386	dehydration-responsive element-binding protein 1D	up
	LOC103344251	dehydration-responsive element-binding protein 1D	up
	LOC103337410	dehydration-responsive element-binding protein 1D	up
	LOC103337406	dehydration-responsive element-binding protein 1F	up
	LOC103332837	dehydration-responsive element-binding protein 2C	up
	LOC103321312	dehydration-responsive element-binding protein 2D	up
	LOC103333423	dehydration-responsive element-binding protein 1B	up
ICE1 transcription factor	LOC103330268	transcription factor ICE1	up
CRPK	LOC103325870	cold-responsive protein kinase 1	up
	LOC103342126	cold-responsive protein kinase 1	up
temperature induced protein	LOC103333219	low-temperature-induced 65 kDa protein	up
	LOC103341239	temperature-induced lipocalin-1	down
LEA	LOC103334143	late embryogenesis abundant protein D-34	up
	LOC103323031	late embryogenesis abundant protein 41	up
	LOC103337622	late embryogenesis abundant protein At1g64065	up
	LOC103337623	late embryogenesis abundant protein At1g64065	up
	LOC103332055	desiccation protectant protein Lea14 homolog	up

Up/down: the regulation pattern in treatment sample.

## Data Availability

Not applicable.

## Supplementary Materials

The following supporting information can be downloaded at: https://www.mdpi.com/article/10.3390/ijms232112831/s1.
