# Supplementary material for: Integrated Transcriptome and Metabolome Analysis of Color Change and Low-Temperature Response during Flowering of Prunus mume
Source: Int J Mol Sci. 2022 Oct 24;23(21):12831. doi: 10.3390/ijms232112831 (PMC9658476; doi:10.3390/ijms232112831)
Supplement: Supplementary file 1 [file ijms-23-12831-s001.zip › Supplementary Figures S1 and S2.pdf]

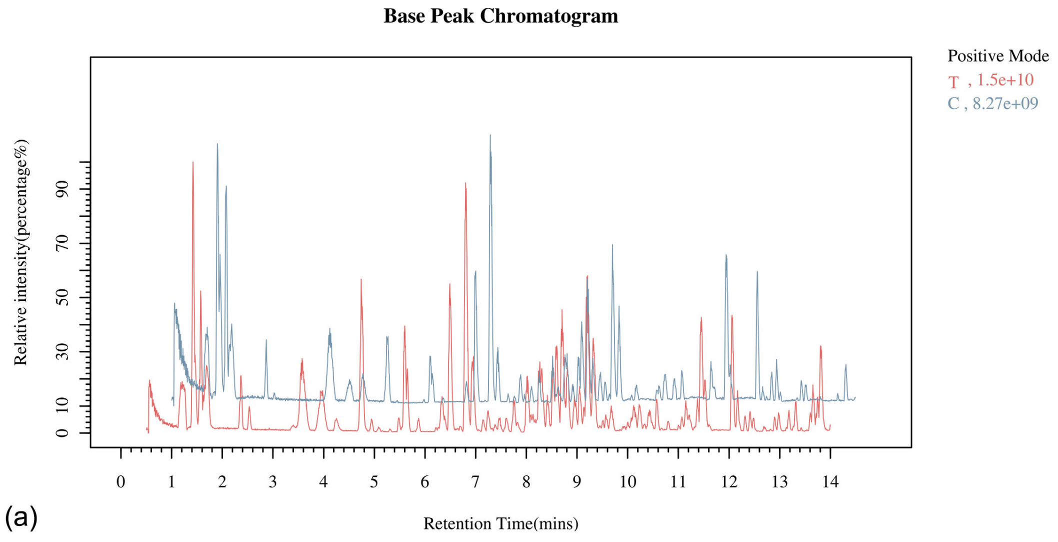

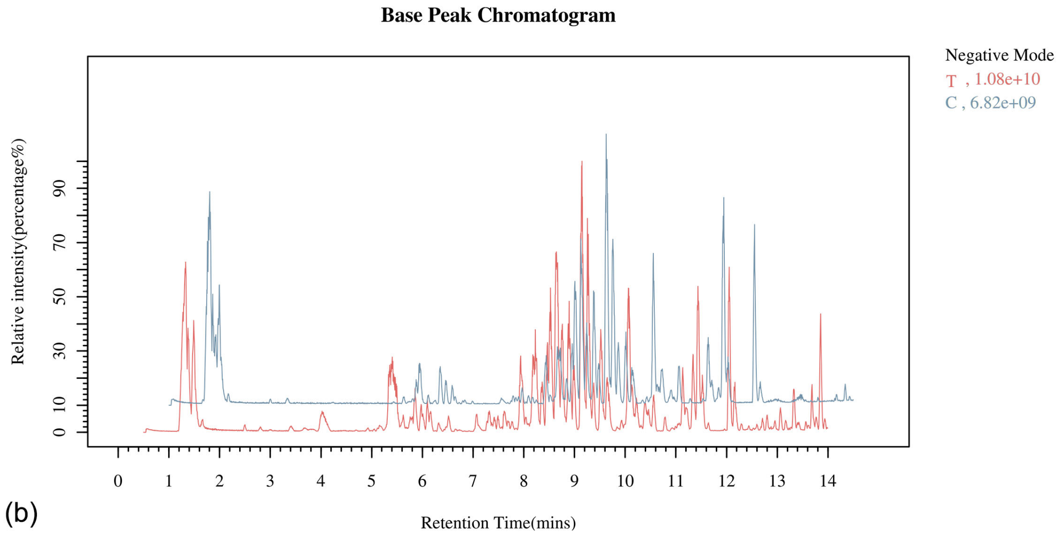

Figure S1. Representative base peak chromatogram of the control and treat groups in the positive ion mode (**a**) and negative ion mode (**b**). Blue represents CK group; Red represents treat group.

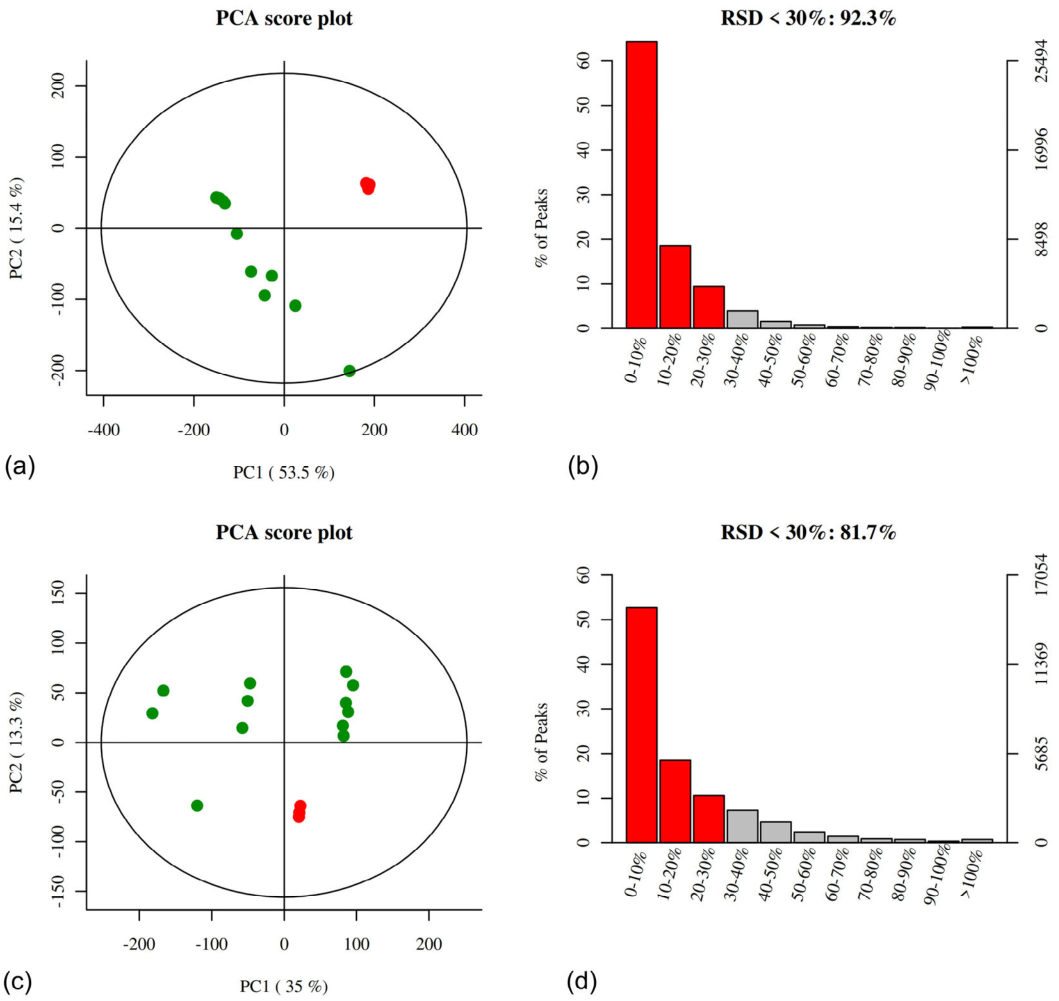

Figure S2. The PCA score plot of the metabolomic data (**a**,**c**), and distribution of the relative standard deviation (RSD) (**b**,**d**) under the positive ion mode and negative ion mode, respectively.
